# An Integrative Model for the Effectiveness of Biofeedback Interventions for Anxiety Regulation: Viewpoint

**DOI:** 10.2196/14958

**Published:** 2020-07-23

**Authors:** Joanneke Weerdmeester, Marieke MJW van Rooij, Rutger CME Engels, Isabela Granic

**Affiliations:** 1 Behavioural Science Institute Radboud University Nijmegen Netherlands; 2 Erasmus University Rotterdam Rotterdam Netherlands

**Keywords:** biofeedback, neurofeedback, anxiety, appraisal, mechanisms, mental health, eHealth, video games, wearable technology, review, mobile phone

## Abstract

Biofeedback has shown to be a promising tool for the treatment of anxiety; however, several theoretical as well as practical limitations have prevented widespread adaptation until now. With current technological advances and the increasing interest in the use of self-monitoring technology to improve mental health, we argue that this is an ideal time to launch a new wave of biofeedback training. In this viewpoint paper, we reflect on the current state of biofeedback training, including the more traditional techniques and mechanisms that have been thought to explain the effectiveness of biofeedback such as the integration of operant learning and meditation techniques, and the changes in interoceptive awareness and physiology. Subsequently, we propose an integrative model that includes a set of cognitive appraisals as potential determinants of adaptive trajectories within biofeedback training such as growth mindset, self-efficacy, locus of control, and threat-challenge appraisals. Finally, we present a set of detailed guidelines based on the integration of our model with the mechanics and mechanisms offered by emerging interactive technology to encourage a new phase of research and implementation using biofeedback. There is a great deal of promise for future biofeedback interventions that harness the power of wearables and video games, and that adopt a user-centered approach to help people regulate their anxiety in a way that feels engaging, personal, and meaningful.

## Introduction

### Background

The era of the quantified self is upon us [1]. Anywhere and at any time, we have access to an abundance of information about how our bodies are adapting to the world around us, be it through the internet, apps on our smartphones, or wearable devices. With these current technological advances, we are able to track, monitor, and regulate various aspects of our physiological and behavioral activation, ranging from heart rate to sleep patterns, to number of steps taken, and calories burned. The popularity of these devices and apps is increasing, and they are becoming increasingly simple to integrate into our daily lives, providing us with the means to take on a more active role in the management of our health and well-being [2-9].

Monitoring our physiological state informs us about not only our physical health but also our psychological well-being, as our physiology is intimately linked with our psychology [10-12]. In particular, our ability to monitor and modulate our emotional arousal, feelings, and expressions—our capacity for emotion regulation—is an integral part of mental health [13,14]. One fundamental aspect of emotion regulation is interoceptive awareness, which is the ability to sense and interpret internal physiological signals [15-18]. Furthermore, physiological dysregulation underpins breakdowns in mental health and psychopathology (eg, anxiety disorders) [19-24]. For some diagnoses such as generalized anxiety disorder and posttraumatic stress disorder, physiological symptoms and dysregulation also serve as important inclusion criteria for diagnosis [19]. However, the majority of current interventions for anxiety and related disorders mainly focus on cognition and behavior, with physiology being seemingly undervalued.

### The Promise of Biofeedback

Together with psychopharmacology, cognitive behavioral therapy (CBT) is currently viewed as the gold standard for the treatment of anxiety [25-27]. Although some components of CBT involve attending to the physiological signals of anxiety, such as self-monitoring and relaxation training, the core of CBT programs is aimed at modifying the maladaptive cognitive and behavioral components of anxiety instead [28-30]. This is an important limitation, as somatic symptoms are strongly related to the severity and level of impairment associated with anxiety [22,31,32]. Therefore, integrating training that facilitates the awareness, monitoring, and regulation of physiological signals might strengthen the effectiveness of anxiety treatment.

In recent years, there has been a rising interest in incorporating self-monitoring technology in the field of medicine and treatment of mental health [7,9,33,34]. In addition to fostering self-regulation, this type of technology also aligns with the current treatment needs as individuals seem to increasingly gravitate toward holistic forms of therapy that emphasize the mind-body connection and allow them to take on an active role in managing their health [7,35]. One promising form of self-monitoring technology is biofeedback. Biofeedback is the process of measuring an individual’s physiological states and feeding that information back to them so that they can learn how to change their physiological activity for the purpose of health improvement [35]. Biofeedback has an extensive history in science [36] and has been shown to be efficacious as a treatment for a large variety of physical [37-39] as well as mental health issues, including stress and anxiety [40-43]. Surprisingly, however, biofeedback has not yet been widely implemented in standard anxiety treatment. Instead, it is generally regarded as an alternative form of treatment [25]. The reported use of alternative treatment is fairly high among people with anxiety, with the most recent numbers indicating that 1 out of 6 (16%) patients with anxiety received alternative care in addition to conventional care [44]. Earlier studies reported numbers ranging between 110 out of 193 (56.7%) [45] and 430 out of 1004 (43%) [26]. However, the available estimate for the specific use of biofeedback among people with anxiety seems fairly small, specifically 3 out of 193 (1.6%) [45].

Part of the reason why biofeedback has not yet been incorporated in standard treatment programs is our general lack of understanding of the mechanisms by which biofeedback training actually works. Although some factors that can explain the effectiveness of biofeedback have been proposed, there is no existing model that encompasses the possible mediating or moderating factors that contribute to positive outcomes of biofeedback training and what should be implemented to strengthen its effects. Understanding the processes that lead to improvement is essential in intervention research, even though they are often overlooked [46]. In addition, practical limitations including hardware requirements, costs, time commitment, and lack of engagement may have prevented wide adaptation [47,48]. However, there have been some promising advancements in recent years that may address some of these limitations, such as the development of biofeedback-based video games, which may increase accessibility and engagement, especially for youth [49-57]. With these current technological advancements in biofeedback training and the accompanying surge in self-monitoring apps, we propose that biofeedback could have a second run in the treatment of anxiety.

### Objective

This viewpoint paper reflects on the current state of biofeedback training for the treatment of anxiety. The theoretical and scientific background regarding the working mechanisms of biofeedback are reviewed. In addition, several key gaps in the empirical literature are highlighted, and reasons for these oversights are addressed. Subsequently, we propose an integrative theoretical model that combines factors that have been traditionally linked to the effectiveness of biofeedback with cognitive appraisals as determinants of the effectiveness of biofeedback training. Finally, a set of guidelines will be presented for future research and the design and implementation of a new wave of biofeedback training. These guidelines will be based on the integration of our theoretical model with the mechanics and mechanisms offered by emerging interactive technologies such as video games and wearables. Using these guidelines, we aim to encourage a new phase of research and implementation using biofeedback.

### Techniques Used in Biofeedback Training

#### Operant Learning

One core technique that is integrated in biofeedback training is operant learning. In biofeedback training, feedback is given about physiological changes that occur. The complexity of biofeedback can vary from presenting raw signals of physiological activity (eg, heart rate variability [HRV], respiration, and electroencephalography [EEG] signals) to a moving frequency analysis [58]. In clinical and educational settings, additional aids are often provided by presenting feedback in the form of graphs, images, or sounds [59]. However, simply showing individuals their activity is insufficient. Instead, it is vital that individuals are actively taught, using operant (or instrumental) learning, how to change their physiological state, specifically by providing real-time feedback and reinforcement while regulation attempts are made [60-64].

In operant learning, positive reinforcement is used to shape an individual’s behavior by strengthening adaptive behavior through the use of rewards, which makes the original behavior more likely to occur [65]. In a typical biofeedback paradigm, an individual is placed in front of a computer screen on which feedback regarding their physiological state is presented. When the appropriate activity increases or inappropriate activity decreases, this change is followed by a pleasant response (eg, a pleasant tone). As sessions are repeated, the thresholds for receiving a reward are gradually modified, thereby stimulating the display of healthy physiological activity [66,67].

#### Meditation Techniques

In addition to operant learning techniques, biofeedback training often incorporates self-regulation techniques that are similar to meditation and mindfulness practices. Meditation is a practice consisting of various exercises that are meant to shift one’s attention primarily to internal stimuli to achieve better well-being and emotional balance [31,68]. Mindfulness meditation likewise aims to shift one’s attention to present experiences, but puts an additional emphasis on regarding experiences in a nonjudgmental and accepting manner [69,70].

Furthermore, similar mechanisms of change can be identified in both biofeedback and meditation. For instance, similar physiological patterns of change are found in response to biofeedback as well as mediation [71], such as a coherent cardiac rhythm [72,73]. Another similarity is that both practices encourage individuals to shift their attention to internal experiences. In biofeedback, this is achieved by using biosensors to provide individuals with feedback on changes in their physiological activity, whereas in mindfulness training, this is achieved by guided mediation exercises where individuals are prompted to shift their attention to the present moment and present experiences [69,70,74]. Both approaches also often train individuals to use slow, diaphragmatic breathing [58,75-79]. However, although both approaches try to enhance individuals’ internal attention, biofeedback specifically focuses on changes in physiological activity, whereas meditation aims for a broader focus on the present moment, including all present experiences. Furthermore, mindfulness meditation explicitly encourages individuals to regard observed sensations in a nonjudgmental manner [69,70], whereas biofeedback training does not. The core difference between meditation and biofeedback, however, is that meditation training does not provide feedback on how the person is doing in terms of regulating their stress. In contrast, biofeedback training gives participants continuous feedback on how they are doing in terms of physiologically regulating their stress. Thus, although there are various similarities between mediation practices and biofeedback, we argue that the feedback provided in biofeedback training is crucial to help people gauge their progress and to keep them engaged in the training.

### Working Mechanisms in Biofeedback Training

#### Interoceptive Awareness

**Figure 1:**
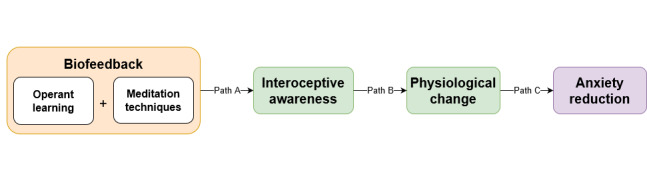
Traditional techniques and mechanisms thought to explain the effectiveness of biofeedback training.

Various mechanisms have been proposed to explain the effectiveness of biofeedback (Figure 1). One of the core assumptions is that biofeedback leads to increases in interoceptive awareness, which in turn helps individuals to better regulate their physiology (Figure 1, paths A and B) [80-82]. Interoceptive awareness is our ability to sense and interpret internal and physiological signals, which is an important part of emotion regulation [13,15,36,83,84]. Scientific research on interoceptive awareness started in the field of psychophysiology back in the 70s and 80s, followed by a new wave of interest in the 90s sparked by the introduction of the somatic marker hypothesis [36,83]. According to this hypothesis, the interpretation of bodily sensations is closely associated with emotional processing and decision making [10,85]. These early days of interoceptive awareness research also gave rise to the idea that biofeedback training could help individuals improve their interoceptive awareness [36], and this assumption is still at the core of biofeedback research today [35,38,41,43,47,59,60,86,87]. The support for this claim, however, seems to be mostly theoretical in nature, as there is very little direct empirical evidence showing that biofeedback training systematically improves awareness of physiological states (Figure 1, path A). Equally important is that it is unclear if and when this internal acuity is adaptive [36,88,89].

Interoceptive awareness has been positively related to decision making [90,91] as well as various forms of emotional processing, such as emotional memory [92], emotion recognition [93], and emotion regulation [15-18]. Importantly, it seems that increased interoceptive awareness is not always better. For example, heightened interoceptive awareness has been linked to increases in anxiety [94,95] and has also been linked to various anxiety disorders such as panic disorder and social anxiety disorder [89,96-99]. In individuals with panic disorder, an increased focus on and the subsequent misinterpretation of physiological sensations is the main cause of panic attacks [96,100]. For individuals with social anxiety disorder, this vigilant attention to physiological sensations likewise impairs their ability to process information from their immediate social environment. Socially anxious individuals often interpret bodily sensations of anxiety as a confirmation that they cannot function well in social situations. Furthermore, these anxious individuals believe that their bodily sensations are clearly noticeable by others, which plays into their fear of being humiliated [89,97,101,102]. Thus, whether or not high levels of interoceptive awareness are adaptive seems to, at least in part, rely on how internal sensations are interpreted.

#### Physiological Changes

Biofeedback training teaches individuals how to regulate their physiological activity [35], and the benefit of biofeedback is often attributed to the acquisition of these self-regulation skills. This belief is rooted in the large body of research dedicated to linking specific emotional states to specific physiological changes [36]. The strong physiological ties of anxiety make it a particularly fitting target for biofeedback, as biofeedback training addresses a wide array of physiological processes. The most prominent forms of biofeedback that are used for anxiety treatment and stress management are HRV biofeedback and neurofeedback.

##### Heart Rate Variability Biofeedback

The primary aim of HRV biofeedback is to create a balance in the autonomic nervous system (ANS). The ANS is divided into 2 branches: the sympathetic nervous system (SNS) and the parasympathetic nervous system (PNS). The SNS prepares individuals for action by increasing their heart rate, blood pressure, and cardiac output, whereas the PNS slows these processes as part of the restorative process [103,104]. Anxiety has mostly been tied to hyperarousal of the SNS, which is associated with physiological responses such as increases in breathing rate and intensity, heightened blood pressure, increases in electrodermal activity, increases in heart rate, and decreases in HRV [42,105]. However, anxiety can also be tied to disrupted PNS function, as is reflected in the low vagal tone that is exhibited by individuals with anxiety disorders [105-109]. Vagal tone is the inhibitory action exerted by the vagus nerve. Specifically, an increase in vagal tone lowers the heart rate and a decrease in vagal tone elevates the heart rate [108]. Vagal tone is used as a marker for emotion regulation, with a higher vagal tone reflecting effective emotion regulation and a lower vagal tone reflecting emotion regulation deficits [110-112].

As the name indicates, HRV biofeedback targets the variability in time (R-R) intervals between heart beats [113,114]. HRV is an important marker for emotion regulation as it reflects the interplay between the PNS and the SNS [110,114-117]. HRV has shown to be particularly sensitive to changes in emotional state, with positive and negative emotions often being readily distinguishable [72]. During negative emotions such as anxiety, there is less synchronization between the PNS and the SNS [118]. In particular, low HRV has been linked to a lack of emotional flexibility [119,120] and has been found to be present in depression [121,122] and anxiety disorders [105,106,109,123].

The primary aim of HRV biofeedback is to create autonomic balance by enhancing cardiac coherence, which is a heart rhythm oscillating at a frequency of approximately 0.1 Hz [39,58,72]. In common HRV biofeedback paradigms, this rhythm is achieved by training individuals to increase the amplitude of respiratory sinus arrhythmia (RSA) [39,58]. RSA is the cyclical change in heart rate occurring when the heart rate increases during inhalation and decreases during exhalation. RSA is commonly used as an index of vagal control of the heart, particularly parasympathetic control [124]. Low amplitudes of RSA are found in anxious and depressed individuals [125,126], and HRV biofeedback aims to increase these amplitudes [58]. In HRV biofeedback training, participants are often shown raw signals of their HRV or a moving frequency analysis. Participants are then instructed to pace their breathing to around 6 breaths per minute to maximize their RSA amplitude [58,75].

##### Neurofeedback

In addition to heart function, emotion regulation deficits in anxious individuals have also been linked to irregularities in electrical activity in various brain regions such as the amygdala and the prefrontal cortex [127,128]. Neurofeedback uses a brain-computer interface to provide feedback to an individual about their brain's electrical activity, which is measured by EEG electrodes placed on the scalp. The aim of neurofeedback training is to maintain the level of brain activity within a specified frequency range (eg, Theta 4-8 Hz) [61,129,130]. Effective neurofeedback training protocols for the treatment of anxiety have mostly focused on enhancing alpha, increasing higher theta activity, and inhibiting beta frequencies [66,67,131,132]. However, quantitative EEG is increasingly used to identify additional patterns of brain activity related to anxiety and to improve neurotherapy (for a detailed overview, refer to the study by Price and Budzynski [132]).

#### Limitations and Gaps in Biofeedback Research

Despite the fact that teaching individuals to change their physiology is central to both HRV biofeedback as well as neurofeedback, there is no convincing evidence as to whether these changes are indeed essential for positive treatment outcomes (Figure 1, path C). In fact, early evidence suggests that physiological changes may only account for a small percentage of variance in clinical outcomes [133,134]. In current efficacy studies, physiological changes are treated as secondary to clinical changes [39], with many studies failing to check whether subjects have been trained to criterion, thus lacking a demonstration that physiological responses have truly been altered [43]. When changes are monitored in neurofeedback, many individuals seem unable to effectively control their physiological activity [135]. However, even in the absence of significant physiological changes, or even when the change was in the wrong direction, significant clinical improvements can still be found [131]. In HRV biofeedback training, similar findings indicated improvements in symptoms of depression and anxiety, although no significant changes in HRV could be established [136]. In addition, improvements in anxiety were found even when sham feedback was given [137]. This could indicate that certain expectations or perceptions of control in relation to biofeedback training may be important determinants for the treatment outcomes of biofeedback [62,135]. However, empirical evidence regarding specific working mechanisms and possible influencing factors related to physiological changes in biofeedback training is lacking.

In the current biofeedback literature, the efficacy of biofeedback is attributed to the implementation of operant learning reinforcement and meditation techniques as well as changes in interoceptive awareness and physiology (Figure 1). Although there is indeed a wealth of theoretical speculation about the importance of these factors, the empirical evidence linking these processes to biofeedback outcomes remains scarce. Equally important is the fact that there are likely important mechanisms that mediate the effectiveness of biofeedback that have not yet been investigated. Specifically, we propose that there is a set of cognitive appraisals that acts as a mediator between previously identified processes (eg, interoceptive awareness and physiological change) and anxiety reduction from biofeedback training. In the section *Cognitive Mechanisms Underlying Biofeedback Processes*, we introduce this set of cognitive mechanisms and discuss the importance of including them in a more comprehensive framework for understanding biofeedback training.

### Cognitive Mechanisms Underlying Biofeedback Processes

Regulating physiology (such as heart rate) is only one aspect of emotion regulation. Emotion regulation also includes changing subjective experience (affective states) as well as cognitions [13]. Cognitive processes, including how we interpret and evaluate certain situations or feelings, play a major role in the onset and regulation of emotion as well as how we respond to and interact with our environment [11,13,138,139]. Evaluations and interpretations of certain events or situations are commonly referred to as appraisals, which are central to our current understanding of emotion [104,140]. Appraisals drive our entire emotional experience, including how our body reacts (eg, sweating or heart racing), how we feel (eg, anxious or calm), and how we take action (eg, avoid or approach) [139,141-144]. Furthermore, dysfunctional interpretations and attributions mediate recovery from mental health problems [145,146]. Appraisals may, therefore, be an important factor explaining or contributing to the effectiveness of biofeedback interventions that aim to help individuals with stress and anxiety.

The broad categories of appraisals include valence (eg, whether something is viewed as positive or negative); causal agency; evaluations regarding one’s potential to cope with or control a situation; and compatibility and relevance with regard to one’s goals, norms, and expectations. Different appraisals of the same situation or stressor can therefore lead to an array of emotional responses [140]. We suggest that there are varying levels of appraisals that are relevant to understanding the mechanisms by which biofeedback works: appraisals of the self, such as self-efficacy and locus of control, and situational appraisals, such as threat-challenge appraisals.

#### Self-Efficacy

When considering appraisal mechanisms by which biofeedback may work to regulate anxiety, appraisals about the self are particularly relevant [140,147]. For example, someone’s uncertainty regarding their ability to cope with a situation is highly predictive of anxiety [148]. Whether someone succeeds in self-regulation may therefore strongly depend on their self-efficacy, in other words, the belief that they can do it. Various meta-analyses have shown that self-efficacy is important for self-development, adaptation, and change [149]. Moreover, high self-efficacy is linked with better emotion regulation skills and general psychosocial functioning [150]. In contrast, low levels of self-efficacy are accompanied by high levels of anxiety [151-153]. In youth, self-efficacy has been found to be predictive of the development and maintenance of affective disorders [154,155]. Furthermore, self-efficacy is an important predictor of treatment outcomes for panic disorder [156]. Given the importance of self-efficacy in anxiety regulation, it is likely a key mechanism for effective biofeedback training.

#### Locus of Control

An additional way in which self-efficacy may be tied to biofeedback is by changing the locus of control. Locus of control refers to the degree to which individuals believe that they themselves have control over the outcome of events in their lives (internal locus) as opposed to forces outside of their control (external locus) [157,158]. People with high self-efficacy generally have an internal locus of control, believing that their own actions and decisions shape outcomes. In contrast, people with low self-efficacy have an external locus of control, often viewing their lives as being beyond their control [149]. Control is an important factor that determines distress and anxiety in a given situation. Specifically, the less someone feels like they are in control, the more anxious they become [159-163]. Moreover, perceived control has been shown to predict outcomes of mental health therapy, with stronger feelings of control being linked to better outcomes [164]. The amount of control someone feels in biofeedback paradigms may therefore influence the success of the training [60,162,164].

Over the course of biofeedback training, with repeated practice and continued feedback on physiological changes, participants in training may increasingly believe that they themselves can influence the outcome of the intervention, in turn leading to a decrease in distress and anxiety. If biofeedback training indeed leads to an increased internal locus of control and locus of control directly influences anxiety, this may also explain why positive therapeutic outcomes of biofeedback can still be present even in the absence of significant physiological changes or when sham feedback is given.

#### Threat and Challenge

Appraisals of the self often interact with more specific appraisals regarding the situation at hand to determine how anxious someone is and whether they are able to regulate their anxiety. For instance, when personal resources are perceived as greater than the situational demands posed by a stressor, the situation is likely to be appraised as a *challenge*. However, when situational demands are perceived as exceeding personal resources, the situation is more likely to be appraised as a *threat* [36,165-167]. Therefore, someone with high self-efficacy is more likely to view difficult tasks as something to be mastered (ie, a challenge) rather than something that should be avoided (ie, a threat) [168].

Challenge and threat appraisals are both related to activation in the SNS. However, they differ in how they prepare the body for action. When individuals enter a threat state, the body responds by activating the hypothalamic-pituitary-adrenal axis, leading to increases in cortisol production. In addition, downstream vascular resistance increases and cardiac efficiency decreases. These changes prepare the body for damage of a physical or social nature. In contrast, when individuals are in a challenge state, there is an increased activation of the sympathetic-adrenal-medullary axis, which results in increased oxygenation of the bloodstream to the brain and peripheral sites and vasodilation, leading to increased cardiac efficiency. These changes prepare the body for approach-oriented behavior [166].

In biofeedback training, the goal is to change a person’s physiological activity from an erratic state to one where there is increased cardiac efficiency and synchrony between the parasympathetic and sympathetic branches of the ANS, that is, a state of autonomic balance or homeostasis [39,58,72,118]. However, the extent to which homeostasis is maintained may not solely depend on practice and feedback but may also be determined or moderated by how physiological activity is appraised over the course of biofeedback training. According to the threat-challenge model, these appraisals are key to changing physiology, cognition, and behavior in stressful situations [166,169]. Indeed, reappraising stress arousal as helpful rather than harmful has been shown to effectively reduce attention bias to threat cues and improve physiological functioning, resulting in decreased vasoconstriction and increased cardiac efficiency [170]. Thus, another way in which biofeedback may work is by helping individuals to shift from interpreting their physiological arousal as being indicative of a challenge rather than a threat.

### An Integrative Model of Biofeedback Training

We began by reviewing the main factors to which the effectiveness of biofeedback has conventionally been attributed, including the implementation of operant learning and meditation techniques and changes in interoceptive awareness and physiology (Figure 1). We suggested that cognitive processes have been largely neglected in biofeedback research even though they play an important role in emotion regulation and adaptive trajectories of anxiety treatment [11,13,138,139,145,146]. Several cognitive appraisal dimensions were identified that may be important determinants of adaptive trajectories within biofeedback training. Specifically, we identified appraisals varying from appraisals of the self to situation-specific appraisals. In the section Real Time Change we propose an integrative model that combines and causally links these appraisals with previously identified mechanisms to explain the effectiveness of biofeedback. In this model, we pay particular attention to both the real-time changes that occur within single biofeedback sessions and the developmental changes that may happen as a result of repeated exposure and training.

#### Real Time Change

**Figure 2:**
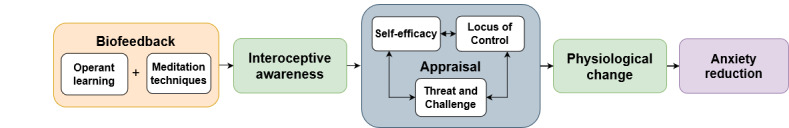
Integrative model of changes in real time including appraisal mechanisms.

Figure 2 summarizes our integrative model of biofeedback effectiveness on a real-time scale. We propose that the relationship between interoceptive awareness and anxiety regulation could be mediated by changes in a person’s moment-to-moment appraisals (Figure 2). Focusing on these real-time processes could potentially address conflicting results in past research on biofeedback. On the one hand, increasing interoceptive awareness through biofeedback is thought to facilitate anxiety regulation. On the other hand, we also know that an increase in physiological awareness can lead to an increase in anxiety [94,95]. Examining how cognitive appraisals may interact in a feedback process in real time with interoceptive awareness helps us make sense of these contradictory findings.

At the start of biofeedback training, participants with heightened anxiety symptoms may begin with low interoceptive awareness, and they may not yet be able to effectively self-regulate. However, as biofeedback training continues, awareness of physiological signals may increase as participants begin to detect changes in their physiology. Importantly, however, this increase in awareness may only have a positive effect on self-regulation when certain appraisals are elicited. For instance, when a person becomes aware of physiological sensations and changes related to their anxiety but they have low self-efficacy, their ability to effectively self-regulate may be impeded and give rise to further anxiety. Alternatively, a person with higher self-efficacy who becomes increasingly aware of changes in their physiology may feel more competent to focus on the training and regulate that physiology. Thus, appraisals such as self-efficacy and locus of control may influence the real-time effects of biofeedback training within individual sessions. However, we argue that for lasting improvements in anxiety to be realized through biofeedback, repeated sessions are needed for locus of control, self-efficacy, and threat-challenge appraisals to change and stabilize into more resilient patterns.

#### Developmental Change

**Figure 3:**
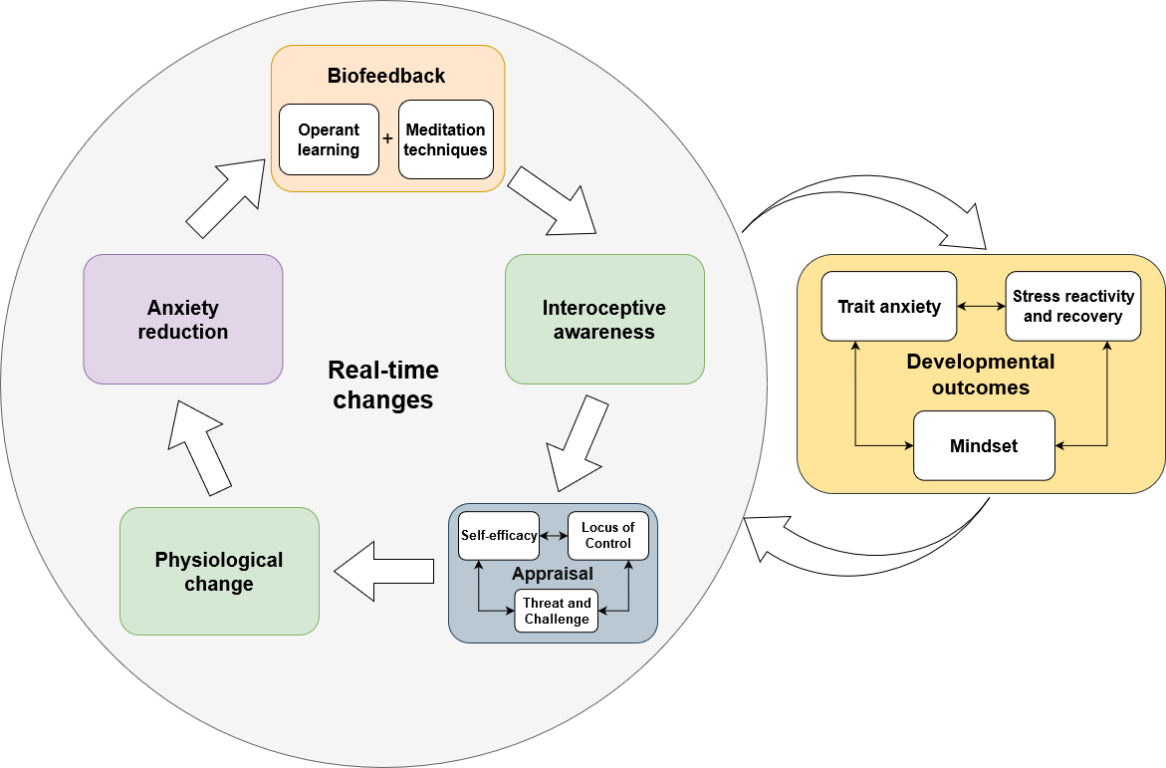
Model of developmental change showing repeated practice leading to changes in mindset, stress reactivity and recovery, and a trait proclivity to feel anxiety.

Some mechanisms of change may already occur within a single biofeedback session, but it is likely that repeated exposure is essential for changes to become automated and internalized. Specifically, we suggest that only when practice is provided in such a way that the proposed changes are optimized will changes generalize to outside the context of the training and influence developmental outcomes such as mindset, stress reactivity and recovery, and trait anxiety (Figure 3).

##### Mindset

Before someone engages in a biofeedback training, they already have certain expectations regarding the efficacy of the training and their ability to change. These expectations are tied to a general belief system regarding the malleability of traits. Someone with a growth mindset believes that traits are malleable and change as a result of circumstances and situations. In contrast, someone with a fixed mindset assumes that traits are stable across time and situations [171,172]. Having a fixed mindset compared with a growth mindset has been found to predict psychopathology [173,174]. Furthermore, a recent study found that students who received a growth mindset intervention viewed stressors as less threatening and showed more adaptive physiological responses, which resulted in better performance outcomes, such as better grades [175]. Differences in individual performance and improvement in biofeedback paradigms may likewise be influenced by someone’s initial mindset with regard to the malleability of self-regulation skills.

Someone with a growth mindset may have a stronger belief that, through practice, they will be able to effectively exert control over their physiology and will regard the training as a positive challenge. This is because individuals with a growth mindset believe change can be obtained through effortful engagement and they regard failure as a sign to remain engaged and bolster one’s efforts [176]. Training as a positive challenge may improve not only someone’s performance in the training but also their physiological functioning [170]. Furthermore, we argue that biofeedback training may lead to changes in people’s mindset. In initial training sessions, participants may start out with a fixed mindset, low self-efficacy, and a low internal locus of control. However, as participants practice repeatedly and observe their progress in terms of self-regulation, they begin to learn that they are indeed able to effectively change their physiology; this awareness, in turn, may influence their mindset. Specifically, someone who previously had a more fixed mindset with regard to their ability to self-regulate could shift toward a more growth-oriented mindset as the training continues.

##### Stress Reactivity and Recovery

Over the course of biofeedback training, participants are also likely to change how they physiologically and psychologically respond to stressful situations (ie, their *stress reactivity*) [177] as well as the speed at which they are able to recover after the stressor has ended (ie, their *stress recovery*) [177]. In particular, anxious individuals exhibit increased stress reactivity and slower stress recovery [177-179], but with repeated practice, they may be able to change their reactivity and increase the speed at which they recover. Specifically, as participants receive practice in changing their physiological activity, they are likely to increase the speed at which they are able to do so. Furthermore, as they receive feedback on their progress, they may change the way they perceive changes in physiological activity and their ability to control this change. For instance, instead of being hypervigilant toward bodily cues and changes in physiology, which often results in increases in anxiety, they may tune their attention to a more balanced, optimal level of engagement. It is likely that *riding* this more balanced form of attention to physiology feels more like a *flow* state [180,181] and results in more positive, agentive, and challenge-based appraisals of the situation. In turn, these challenge-based appraisals will improve stress reactivity and may help individuals to recover more quickly from being exposed to stressors [166,169,170].

##### Trait Anxiety

With repeated practice, as the self-regulation techniques and changes in cognitive mechanisms become more internalized, people may become more adept at appraising and regulating their physiological and psychological responses to stressful situations. Over development and repeated training sessions, we propose that an increased growth mindset and more adaptive stress reactivity and recovery will develop in concert with lower day-to-day levels of anxiety (ie, their trait anxiety).

### Implications for Research

Studies testing the effectiveness of biofeedback training have primarily focused on outcome measures. However, to update theoretical models that link physiology to emotional states, and to improve outcomes from biofeedback interventions, it is vital to study how individuals change with repeated practice and exposure and how outcomes may be tied to certain mediators and moderators. In particular, there seems to be a great deal of promise in studying the appraisal processes outlined in Figures 2 and 3 as mediators within individual sessions as well as across sessions to determine how they influence developmental outcomes.

To capture the mechanisms of change, experimental studies need to be set up in such a way that measurements of interest are repeated within and across multiple training sessions. For instance, training at the very beginning may make individuals feel like they do not have a lot of control over their physiology and the way that they feel, but this perceived control may increase over the course of the training. By assessing locus of control at multiple times during the training, the extent to which changes in these appraisals, in real time, predict or perhaps mediate the effect of the training on anxiety symptoms can be examined. Subsequently, by examining at what point in the training and which particular circumstances these changes occur, future training programs can be designed to amplify these changes and subsequently maximize positive outcomes.

Although it is important to capture change, it is also important to examine how certain beliefs or characteristics present before starting the training (ie, cognitive moderators) may influence performance or how quickly someone learns to self-regulate. For example, someone with a growth mindset who believes that they are able to change their ability to self-regulate through practice may be more receptive to feedback about their physiology, viewing the training as an opportunity to better themselves. In contrast, a fixed mindset may initially inhibit progress in the training, as physiological sensations of anxiety and feedback on physiology may be interpreted as threatening. Thus, in studies focused on evaluating the effect of a biofeedback training, it seems important to include mindset as a moderator in the analyses. Alternatively, participants can be divided into groups using a particular cutoff of the characteristic of interest. The extent to which individuals with one particular trait (such as growth mindset) improve more than those with a fixed mindset can be examined. It would also be interesting to track the trajectories of change over the course of the various training sessions to determine whether they differ between groups. Information about the influence of certain traits and characteristics can inform us about who is an ideal participant for biofeedback training and why, such that future interventions can be improved and better targeted. For instance, if the results indicate that individuals with a growth mindset respond better to biofeedback training, then it may be more effective if the training is adapted in such a way that expectations related to this type of mindset are primed early on in the intervention.

### Implications for Designing New Biofeedback Interventions

Thus far, we have proposed a new integrative model that presents several cognitive appraisals as possible moderators or mediators of adaptive outcomes in biofeedback training and have suggested ways in which these appraisals can be assessed in future experimental studies. Furthermore, we emphasized that adaptive and lasting developmental changes can only be achieved by repeated practice. Our design recommendations follow from these points, emphasizing 2 main sets of suggestions. First, biofeedback interventions should work optimally if they enforce all factors in our model, including interoceptive awareness and physiological change as well as the newly proposed cognitive mechanisms. Second, these interventions need to be designed to keep individuals intrinsically motivated and engaged enough to keep practicing over several repeated training sessions. In the following sections, we describe how specific design aspects of interactive technology, such as video games and wearables, could be integrated with biofeedback training to optimize the factors described in our integrative model.

#### Interoceptive Awareness

Even if someone may become increasingly aware of their internal state while engaging in biofeedback training, it may still be difficult to notice changes in physiology in someone’s daily life. Although continued practice may increase the likelihood that changes and skills transfer to outside the training context, this process may be further enhanced by using wearable self-monitoring technology. *Wearable* technology is becoming increasingly easy to integrate in our daily lives [9]. There are already a large number of mobile phone apps available that are either linked to internal sensors (eg, global positioning system or pedometer) or external sensors (eg, heart rate sensors), which can generate real-time information or provide daily, weekly, or even yearly overviews of a person’s activities, mood, and physiology. Using these wearables may not only increase someone’s interoceptive awareness but may also provide information regarding the contexts in which certain internal changes are most likely to occur. Furthermore, providing an overview of how someone has changed over a certain period may foster a growth mindset as it emphasizes their malleability. Finally, reports provided by wearables may also be valuable for clinicians as they can more closely monitor their clients’ improvement or deterioration and identify contexts in which certain exercises or interventions may be most effective.

At this point, it is important to emphasize that although interoceptive awareness is an important aspect of emotion regulation, merely increasing this awareness may actually result in an increase in anxiety [94,95]. Continuously providing feedback on whether a desired level of physiological activity is reached may lead to extreme attention vigilance and be counterproductive for those anxious people who already focus too much on their internal states. Therefore, fostering a balanced awareness may be a better approach for biofeedback training for anxiety reduction. To achieve this balance, the training should be designed in such a way that other elements in the environment provide opportunities to disengage from attending to physiological activity. For instance, in the biofeedback video game Dojo [49-51], there is a figure of a seated human with a heart displayed in the corner of the computer screen that changes color based on the player’s HRV. Specifically, the heart changes in color from red to orange to green, with green being the most optimal level of HRV. Using this type of stimulus clearly communicates changes in the physiology of the player; however, having it continuously present may result in a singular focus on their internal state. Thus, making the training context more engaging in different parts of the game and leading participants’ attention away from the heart representation for short periods is likely a more optimal strategy. It may actually be most effective to move away from explicit representations of heart rate, pulse, and other bodily representations of stress and arousal.

If the digital context where self-regulation is practiced is engaging enough (eg, by offering thrilling or beautiful environments to explore where stimuli respond to changes in physiology), these design features may craft a more balanced attention landscape. Practicing in this type of landscape may change from a vigilant form of interoceptive awareness to a state of concentrated relaxation in which participants are aware of their arousal, but instead of fighting it or losing themselves in it, they are in control and move along with it. Gamers have described this state of *concentrated relaxation* as feeling completely focused and concentrated but at the same time feeling like they are able to let go of both [182,183]. For example, the game Nevermind [52], in addition to using explicit representations of HRV (a heart changing in color), also uses atmospheric changes that reflect the player’s level of arousal. Players find themselves in an eerie environment that becomes more or less unsettling depending on how well the player is able to self-regulate their arousal. The screen becomes more distorted or ominous noises become even more pronounced as the person feels more anxious. Moreover, specific scenic elements are used, such as the kitchen that floods with milk, the more anxious the player becomes. In addition, the game also includes challenging puzzles that require the player’s attention, which means they cannot singularly focus on changes in their HRV [53]. Another exemplary biofeedback app is DEEP, which integrates atmospheric forms of biofeedback [54]. In DEEP, players receive feedback on their breathing by means of a circle that enlarges and shrinks in accordance with the player’s breath. Although this circle is always directly in the player’s line of sight, the game environment is designed in such a way that it grabs the attention and invites players to explore the beautiful underwater world with its vibrant flora and fauna that respond to players’ breath. For example, some of the plants mirror players’ in and out breath by growing and shrinking or becoming more or less bright [55,56]. As these examples demonstrate, there is a great deal of promise in integrating biofeedback into engaging environments where participants are made aware of their internal states but feel competent and able to focus on the task at hand.

#### Self-Efficacy and Locus of Control

One of the foremost sources of information from which we derive our sense of self-efficacy is the genuine experience of mastery [184]. Specifically, our feelings of competence and confidence are largely based on earned failures and successes [184,185]. A sense of mastery can be felt when a task’s level of challenge is aligned with our perceived level of competence. In the developmental literature, this ideal balance between challenge and guidance is known as the zone of proximal development [186]. Putting players in this sweet spot of challenge to keep them motivated and engaged is something that video game designers excel at, as is demonstrated by the large number of individuals that play games and by the number of hours that they play them [187-189].

This balance of challenge and difficulty experienced in games is also referred to as a state of flow. Flow is experienced when individuals are immersed in a task, with energized focus, full involvement, and enjoyment in the process of the activity. This flow state coincides with a high sense of control and a loss of self-consciousness [181]. Notably, flow has also been linked to increased self-esteem and decreased anxiety [190]. To achieve a flow state, clear goals must be set beforehand and there must be a possibility to monitor progress. In addition, clear and immediate feedback needs to be given with the aim of balancing the perceived challenge and one’s perceived skills [180]. Some of these conditions are already an inherent part of biofeedback, such as immediate feedback and the ability to monitor progress. However, most biofeedback training programs are not specifically designed with flow states in mind. Video games are designed in such a way that flow states are likely to occur as the difficulty level dynamically adapts to the player’s current level of mastery, ramping up whenever the skill of the player increases [191]. Although some biofeedback programs do in fact dynamically adjust the level of difficulty, participants may still not experience mastery. To accomplish a true sense of mastery, it is not only important to balance the level of challenge but also to balance rewards and acknowledgments of success. Providing rewards when appropriate instils a sense of accomplishment and pride. However, if challenges are too easily overcome and rewards are disproportionately doled out, the same sense of accomplishment is not felt due to ill-gotten gains [192]. When designing biofeedback interventions, it may therefore be beneficial to focus on facilitating flow and genuine mastery experiences, thereby creating experiences where participants feel competent, in control, and focused on the goals in the training but are still aware of their physiological arousal.

#### Threat and Challenge

Clinical interventions often focus on decreasing physiological arousal as increases in arousal (eg, quickening of the breath and an increase in heart rate) are often associated with negative emotional states such as anxiety. Interventions that focus on changing appraisals of stress from a threat to a challenge do not focus on reducing arousal or inducing relaxation per se, but instead aim to change the way the arousal is interpreted and experienced [169,170,193]. Interventions aimed at changing threat-challenge appraisals either directly target arousal interpretations or target higher-level belief systems such as growth mindset. When directly targeting threat-challenge appraisals, participants are often provided with information in a written, oral, and sometimes video form, which demonstrates that arousal is not harmful but rather a functional response of the body that aids performance in challenging and stressful situations. Interventions focused on fostering growth mindset use similar strategies but have an additional emphasis on people’s ability to change (for a complete review of threat-challenge manipulation strategies, refer to the study by Jamieson et al [193]). Appraising arousal in a positive manner has been shown to lead to more adaptive stress responses [166] and even better academic performance [169,175].

To encourage challenge appraisals, feedback messages could be implemented in biofeedback training before and throughout biofeedback training, which frames arousal as adaptive. However, a downside to integrating these types of messages is that they are fairly explicit, which may clash with the more implicit, bottom-up type of feedback that is delivered through biofeedback. Therefore, if feedback messages are integrated, they should nicely blend into the environment. One successful example of this type of integration can be found in the phone-based companion app called #SelfCare [194]. In this app, which aims to provide relaxation and mindfulness training, users are invited to stay inside their virtual bedroom where they can interact with objects in the environment and play some minigames. When interacting with the app, messages pop up on screen that encourage the user to observe how they feel, such as “Is there tension somewhere?,” “Where does this tension come from?,” and “What can we learn from it?.” These types of messages frame bodily sensations and arousal as informative, similar to threat-challenge manipulation strategies [193]. Furthermore, the user is addressed in a comforting and encouraging way, such as “There’s no hurry, we can stay as long as we’d like.” However, the messages do not feel patronizing or forced because they are phrased in a personal and relational way and because they blend in with the soothing and safe atmosphere of the environment. Thus, although biofeedback training may benefit from integrating feedback messages that frame arousal as functional and encourage a growth mindset, we argue that caution must be given so that it is well balanced with the design.

In previous sections, we posited that merely interacting with biofeedback could already help individuals to appraise increasing arousal from a perspective of challenge as they witness that arousal as something they can control. However, the environments in which participants train are often fairly neutral, and participants are rarely given the opportunity to practice in situations that increase their arousal [57]. We argue that especially when it comes to anxiety, it is important that individuals practice in contexts where feelings of anxiety are actually triggered, similar to exposure exercises in anxiety treatment [195]. When practicing self-regulation within contexts that evoke arousal, the likelihood increases that skills are transferred outside of the training to stressful situations where these skills are needed most. Moreover, when practicing within these contexts, reappraisals of arousal are more likely to occur than practicing in a neutral or relaxing setting [196,197]. Some biofeedback-based games such as Nevermind [53] and Mindlight [198] have already adopted the use of anxiety-inducing settings. The latter game, Mindlight, has also been shown to be as effective in relieving anxiety as CBT [199]. Ideally, stressful training environments are designed to increase arousal, but they also allow individuals to directly interact with and exert control over the environment so that they feel immersed, in control, and motivated to continue.

#### Mindset

Although biofeedback training may already implicitly change participants’ mindset as feedback with regard to change and progress is constantly provided, incorporating design elements that explicitly facilitate a growth mindset may further strengthen the training’s effects. Various approaches have been used in previous interventions to foster a growth mindset, such as providing individuals with excerpts from scientific texts, showing them educational videos, or giving them writing exercises. All these strategies focus on priming individuals with or explicitly displaying information that emphasizes people’s ability to change [175,200]. These mindset interventions have shown positive results in improving physiological responses, work performance, affective responses, and health outcomes (for overview, refer to the study by Jamieson et al [193]).

Although biofeedback training may already foster a growth mindset by providing feedback with regard to physiological change, the training may further benefit from including elements that are explicitly designed to emphasize a person’s malleability. For instance, feedback messages in initial biofeedback sessions could be adapted so that they not only indicate how someone is currently doing but also highlight their rate of improvement and that they have the potential to further improve. Furthermore, participants can be provided with regular opportunities to check their progress throughout the course of the training. For instance, they could be shown a visual representation of all changes that they went through from the first session onward. Specifically, feedback messages in the initial session can be used to demonstrate the possibility of change, and showing explicit evidence of a person’s progress can serve as a more concrete representation of improvement. Furthermore, for these growth mindset microinterventions, it is essential to structure the specific feedback messages such that they reinforce and focus on the participant’s efforts instead of emphasizing that the participant is a competent person [176]. For instance, messages should not focus on personal traits such as “You’re awesome!” or “You’re so smart!” Instead, these messages should highlight progress and effort such as “You’re doing great, it’s clear that you are improving!” or “Keep at it, you’ll figure it out!” Adding these types of feedback messages could result in someone adopting a growth mindset regarding their ability to self-regulate.

### Engagement as a Prerequisite for Change

In the previous section, we have shown how future biofeedback interventions can be designed in such a way that they optimize all factors in our integrative model (Figure 3). Several of these suggestions focused on harnessing the power of new technologies such as video games and wearables. These forms of technology can directly target some of the suggested mechanisms and are also more likely to keep users engaged and motivated. However, although we have proposed that using these forms of technology is promising in terms of accessibility and engagement, we also suggest that merely integrating game elements or wearables into a biofeedback intervention is not sufficient to guarantee user engagement. For instance, the use of wearables has increased in recent years, and one-third of all adopters stop using their devices after a couple months [201]. A similar lack of adherence and uptake has been observed for the use of digital mental health apps, including game-based interventions [202-205]. For wearables, the observed drop in usage is attributed to the fact that many of these apps suffer from poor user research (or none at all), which results in unsatisfactory user experiences [7,206]. A similar lack of user-centered approaches and design principles has been posited for digital mental health interventions [202,204].

One possible solution to avoid the same pitfalls in the design of the next generation of biofeedback interventions may be to adopt design thinking principles and practices. A recent paper by Scholten and Granic [204] outlined how design thinking could be used to improve digital mental health interventions. Design thinking focuses on empathy, multidisciplinary ideation, and experimentation. The design thinking approach puts the needs of the user at the center of the development process. Furthermore, there is cross-disciplinary teamwork and collaboration, and the development process consists of rapid prototyping and iterative testing of services [204]. Although this is just one suggested approach, its emphasis on user engagement integrated with a strong design foundation that tailors the intervention or app to the user’s needs seems particularly promising.

### Conclusions

With current technological advances and the increasing interest in the use of self-monitoring technology to improve mental health, we argue that this is an ideal time to launch a new wave of biofeedback training. Our hope for this paper was to inspire a new phase of research and implementation of biofeedback training. We reviewed the more traditional techniques and mechanisms thought to explain the effectiveness of biofeedback: operant learning and meditation techniques, interoceptive awareness, and physiological change. We then identified several cognitive appraisal dimensions as potential determinants of adaptive trajectories within biofeedback training, including self-efficacy, locus of control, and threat-challenge appraisals. Subsequently, we proposed a new comprehensive model addressing real-time as well as developmental processes of change. Specifically, we posited that the relationship between changes in interoceptive awareness and anxiety regulation in individual sessions may be mediated by changes in a person’s moment-to-moment appraisals such as self-efficacy and locus of control. Furthermore, we highlighted the importance of repeated exposure and practice to achieve adaptive and lasting developmental changes in growth mindset, stress reactivity and recovery, and trait levels of anxiety. Finally, we presented guidelines for the design of future experimental studies as well as new biofeedback training programs and apps that are in line with our integrative model. In summary, there is a great deal of promise for future biofeedback interventions that harness the power of wearables and video games and that adopt a user-centered approach to help people regulate their anxiety in a way that feels engaging, personal, and meaningful.

## Abbreviations

ANS: autonomic nervous system
CBT: cognitive behavioral therapy
EEG: electroencephalography
HRV: heart rate variability
PNS: parasympathetic nervous system
RSA: respiratory sinus arrhythmia
SNS: sympathetic nervous system
